# Household satisfaction with community-based health insurance scheme and associated factors in piloted Sheko district; Southwest Ethiopia

**DOI:** 10.1371/journal.pone.0216411

**Published:** 2019-05-13

**Authors:** Kindie Mitiku Kebede, Sharew Mulugeta Geberetsadik

**Affiliations:** Department of Public Health, College of Health Sciences, Mizan -Tepi University, Mizan-Teferi, Ethiopia; University for Development Studies, GHANA

## Abstract

**Background:**

Community-based health insurance (CBHI) scheme is an emerging strategy for providing financial protection against health-related poverty. It is being piloted in the Sheko district, but community satisfaction with the scheme has not yet studied. Therefore, this study aimed to assess the level of household’s satisfaction to CBHI scheme and associated factors in a piloted Sheko district; southwest Ethiopia.

**Methods:**

A community-based cross-sectional study was conducted in Sheko district from March to April 2018. Data was collected on 528 households by using simple random sampling method. Trained data collectors gathered the data using a pre-tested and structured questionnaire. Descriptive statistics, bivariate and multivariable logistic regression analyses were performed. To determine the independent predictors of household’s satisfaction to CBHI, a cut point of p values < 0.05 were used.

**Results:**

This study showed that more than half (54.7%) of the households were satisfied with the CBHI scheme. Satisfaction to CBHI was positively associated with adequate knowledge of CBHI benefit packages (AOR = 2.29, 95% CI = 1.55–3.38), type of health facility visit (AOR = 1.93, 95% CI = 1.09–3.39), laboratory service provision (AOR = 2.07, 95% CI = 1.15–373) and length of enrollment (AOR = 1.53, 95% CI = 1.01–2.32).

**Conclusions:**

Household’s satisfaction to CBHI scheme was moderate. Modifiable factors, including adequate knowledge of CBHI benefit packages, type of health facility visit, laboratory service provision, and length of enrollment were independent determinants of satisfaction. In order to augment enrollee’s satisfaction to CBHI, efforts should be given to improving their knowledge of CBHI benefit packages through education and information campaigns. Furthermore, due consideration should also be given to improving the quality of health services.

## Background

Low-income countries (LICs) face substantial challenges in financing healthcare [1, 2]. Health services are unaffordable and even unavailable to the majority of poor people in these countries [3]. Health spending via out-of-pocket payments (OOPs) is difficult for many people and millions of people fall into poverty due to the need to pay for healthcare [4]. As a result, the poor people in LICs still suffer and die from health-related problems particularly in settings that lack effective health insurance policies [5].

In Ethiopia, easily preventable communicable diseases are still a major public health problem [6]. However, health seeking behavior and access to modern health care is low in rural areas[7, 8]. One of the reasons for low utilization of modern health care services is the user fee charges [9]. User fees can present a substantial psychological and financial burden to the families. It is one of the barriers to healthcare use, especially for poor households who are themselves likely to be particularly vulnerable to ill health [10]. Thus, moving away from out-of-pocket charges for health care at the time of use is an important step towards averting the financial hardship associated with paying for health care service [11].

To overcome financial hardships associated with out-of-pocket payments, the Ethiopian government has introduced two types of health insurance schemes since 2010 [12]. The first kind is CBHI and the other one is social health insurance (SHI). The SHI intends to cover 10.46% of the population who are engaged in formal sectors. CBHI, on the other hand, intends to cover 83.6% of the populations of Ethiopia who are engaged in the informal sectors.

The government of Ethiopia is working to narrow the existing wide gap between community demand for health care and financial constraints in the health sector by implementing the CBHI scheme in rural areas [13]. Currently, CBHI scheme is being implemented over 161 districts and recent evaluation shows improvements in health service utilization among districts implementing CBHI scheme[12].

CBHI enrollees expect better quality of care. Thus, providing better quality of care is crucial for client satisfaction and sustainability of the CBHI scheme. A study finding in India shows that there was high level of satisfaction among insured clients than uninsured [14]. However, from a practical point of view, CBHI enrolment status alone could not be a guarantee to get quality health care services. For instance, Robyn PJ and colleagues found that insured people objectively receive the worse quality of care than uninsured people in Burkina Faso[15].

Though the health insurance satisfaction study is an ongoing, some studies show that level of satisfaction varies from region to region [16–18]. A study in Nigeria shows that less than half (42.1%) of enrollees were satisfied with health insurance scheme in 2011[17]. However, a high level (91.38%) of household’s satisfaction to CBHI was reported in Ethiopia in 2016 [18].

Several studies show that socio-demographic characteristics such as sex, age, marital status, educational level, occupation, and family size affect enrollee’s satisfaction to health insurance [16–18]. In addition to socio-demographic factors, health service related factors also influence enrollee’s satisfaction to CBHI[18]. Satisfaction to CBHI is positively associated with enrollee’s perception of good laboratory service provision; health provider’s friendliness; availability of doctors and medicines [14, 18]. Furthermore, studies in developing countries have shown that satisfaction of the health insurance scheme is influenced by the enrollee’s knowledge of health insurance benefit packages[17, 19].

CBHI scheme has been piloted in this study area since the end of 2016. However, enrollee’s satisfaction and contributing factors are not yet known. Hence, whether this scheme has brought quality health care and enrollees have a positive perception towards CBHI scheme (measured by client satisfaction) is unknown so far in this study area. Up to the level of our knowledge, only one study was conducted to assess household’s satisfaction to CBHI scheme in another part of Ethiopia [18]. As quality of care varies from one area to the other, the level of satisfaction and contributing factors also varies from one context to the other.

Therefore, the aim of this study was to assess the level and associated factors of household’s satisfaction to the CBHI scheme in order to recommend policy makers and programmers to improve satisfaction and acceptance of CBHI scheme by the communities.

## Methods

### Ethical approval and consent to participate

Ethical clearance was obtained from Mizan-Tepi University research directorate. The aim and potential benefits of the study were discussed with all of the participants. Informed written consent was taken.

### Study design and setting

A community-based cross-sectional study was conducted starting from March to April 2018. This study was conducted in Sheko district; one of the two districts selected for the CBHI pilot project in Benchi-Maji zone. Sheko district is located in Benchi-Maji zone, Southwestern region of Ethiopia and 580 KM away from Addis Ababa. The district has 24 rural kebeles (the lowest administrative unit in Ethiopia) and an estimated total population of 65,554 in 2018.

Sixty one percent of the households in the district were enrolled in the CBHI pilot scheme. The district has 3 health centers and 24 health posts. The health coverage of the district was 100% in 2018[20].

### Study population

All CBHI users in Sheko district were the source population. Sampled CBHI scheme users that were interviewed during the study period were considered as study population and the study unit was household heads (be it female or male).

### Inclusion and exclusion criteria

Households that had at least one family member who visited public health facilities at least once starting from their enrolment in CBHI scheme were included. Participants (household heads) who were seriously sick and unable to give response were excluded.

### Sample size determination

The sample size was calculated using single population proportion formula (Zα/_2)_^2^*p(1-p)/d^2^ assuming 91.38% of households satisfied with CBHI scheme taken from a study conducted in Ethiopia [18], a confidence level of 95% and a 0.03 margin of error.

We followed a two-stage sampling procedures. To account this, the sample size was multiplied by the design effect of 1.5. Finally by adding 10% non-response rates, the final sample size was 528.

### Sampling techniques and procedures

A two-stage random sampling technique was employed to select the study participants. We applied simple random sample selection at each stage to eliminate selection bias. In the first stage, 30% of kebeles were selected using a lottery method.

**Fig 1:**
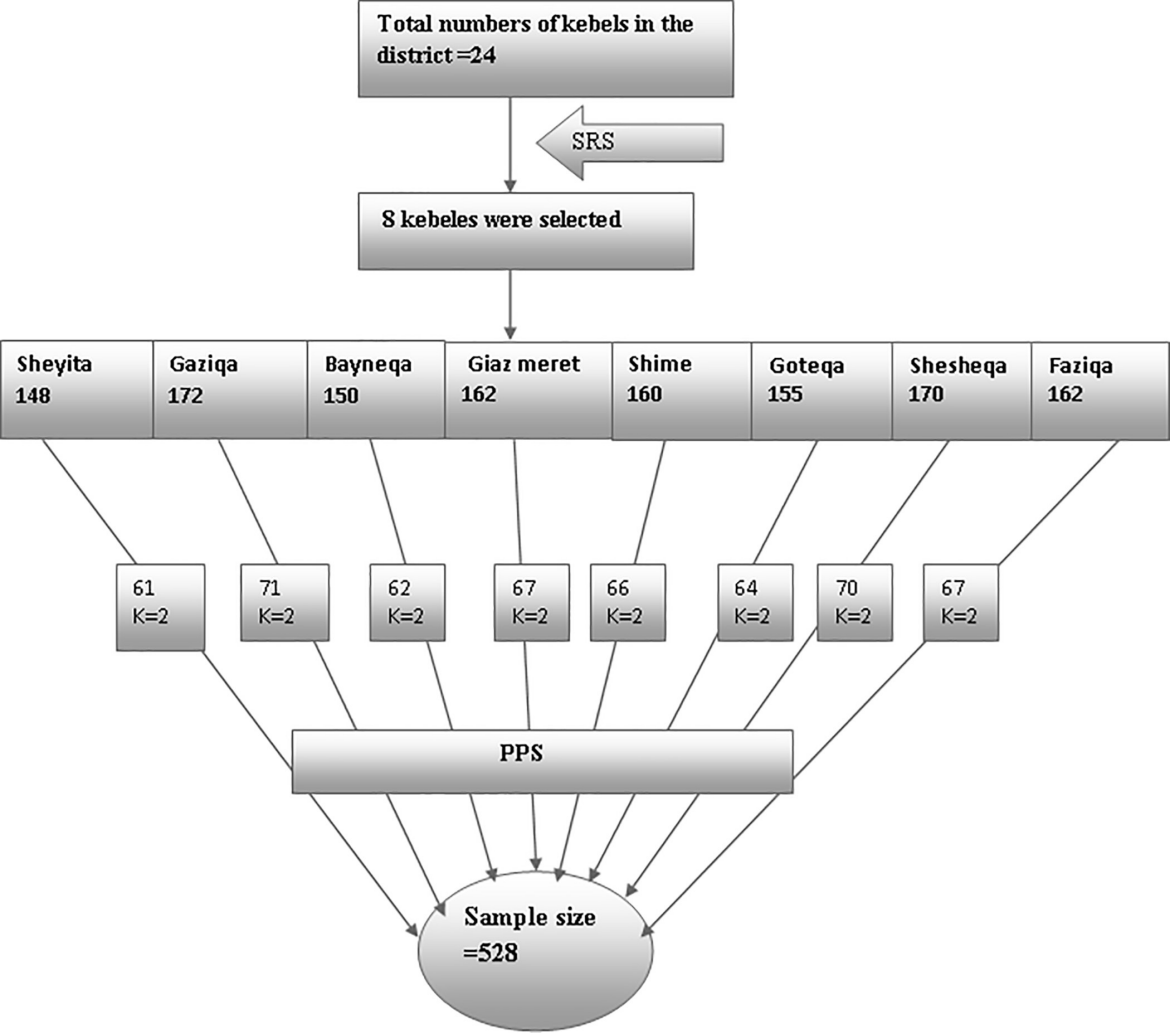
Sampling procedure of study participants in the CBHI piloted Sheko district, Southwest Ethiopia, 2018. SRS = simple random sampling; K = sampling interval; PPS = probability proportional to size.

In the second stage, households enrolled in CBHI in the selected kebeles were identified using their individual enrolment identification numbers from the registration book through the help of health extension workers. Then, the sample size was proportionally allocated to each kebele. Finally, the study participants were selected using systemic sampling methods. The sampling fraction was determined by dividing the proportionally allocated size to the total sample size. The overall sampling procedure is presented in **Fig 1.**

### Data collection and quality assurance

A pretested and structured questionnaire developed from various literatures [14, 16–18] was used. The questionnaire was prepared in English and translated from English to Amharic language (S1 File). The interviews were conducted face-to-face by diploma holders who are fluent in Amharic and local language.

Before data collection, a pretest was conducted in Debub-Benchi district among 5% of the sample size. Based on the pretest results, adjustments were made to the data collection tool. Spot checks on the quality of data collection were made in the field and completed questionnaires were checked daily. Data collectors were given 2 days training on the study objectives, method of data collection, and the tools for data collection. Senior public health professionals were recruited to supervise data collectors. A second visit was made for households closed during the data collection period. Households closed during the second visit were considered as non-respondents.

### Data analysis

Data were entered into EpiData version 3.1 and exported to SPSS version 20.0 for further analysis. The frequency distributions of all the variables were examined to check data entry errors. Descriptive statistics such as frequencies, medians and percentages were calculated as univariate analysis. Bivariate analysis was carried out to assess the association of each independent variable and household satisfaction to CBHI scheme.

Multivariable logistic regression model was fitted to control confounds and ascertain the independent predictors of household satisfaction to CBHI scheme. The logistic regression model was statistically significant x^2^(12) = 58.85, p<0.001 and explained 63.5% of the variance of household satisfaction to CBHI scheme. The Hosmer & Lemeshow goodness of fit test was insignificant (p = 0.45) which indicates that the model was adequate. To assure the reliability of the tools used in this study area, Cronbach’s alpha coefficient was calculated for the outcome variable (household satisfaction to CBHI scheme). Variables with a p value of less than 0.2 in the bivariate analysis were included in the multivariable logistic regression analysis.

Variables with p values <0.05 at the multivariable analysis were considered as significantly associated with CBHI scheme.

### Measurements

#### Household head’s overall satisfaction

The household head’s overall satisfaction to the CBHI scheme was considered as an outcome variable. Nine items related to satisfactions on a five point Likert scale from strongly disagree to strongly agree were used. The nine items were: i) local CBHI management is trustworthy; ii) satisfied with the opening hours of the CBHI; iii) satisfied with the collection process of insurance cards; iv) satisfied with the time to make use of the CBHI program after payment of registration fee; v) satisfied with the schedule for paying of premium; vi) satisfied with the information provided; vii) satisfied with CBHI packages; viii) want to stay enrolled in the CBHI scheme and; ix) recommending CBHI scale up to other settings. Then, households were leveled as satisfied if their response was ≥ median score of satisfaction questions otherwise it was leveled as not satisfied.

#### Knowledge of CBHI benefit packages

Households were asked seven items related to the CBHI benefit packages. The seven items were the followings:i) CBHI is a good way of helping clients to health expenditure; ii) CBHI covers only care from public health institutions; iii) CBHI covers only care within the country; iv) CBHI doesn’t cover transportation fee; v) CBHI covers outpatient care; vi) CBHI covers inpatient care and; vii) CBHI doesn’t cover medical care for cosmetic values. Then, households were labeled as having adequate knowledge if answered more than four of the CBHI benefit packages. Otherwise, households were labeled as not having adequate knowledge of CBHI benefit packages.

## Results

### Socio-demographic characteristics of the participants

Five hundred twelve household heads were participated in this study yielding a response rate of 96.7%. Of these, 391 (76.4%) were male. The median age of participants was 35 years old. The majority of respondents (83.6%) were married to one spouse. Nearly half (50.6%) of the households had greater than 5 family members and the median family size was 6 members. About 209 (40.8%) of participants didn’t attend formal education. The estimated median family income per annum as reported by household heads was 8,000.00 Ethiopian birr (Table 1).

**Table 1:** Socio-demographic characteristics of the participants enrolled in a piloted CBHI scheme in Sheko district, Southwest Ethiopia, 2018 (n = 512).

Variables	Frequency	Percent
**Household head’s Age** Median age = 35 years old		
<30	130	25.4
30–39	230	44.9
40–49	105	20.5
≥50	47	9.2
**Sex of household head**		
Male	391	76.4
Female	121	23.6
**Marital status**		
Married to one spouse	428	83.6
Married to more than one spouse	48	9.4
Unmarried	36	7.0
**Religio**n		
Christian	492	96.1
Muslim	20	3.9
**Family size** Median = 6(IQR = 3)		
1–5 members	253	49.4
>5 members	259	50.6
**Household head’s educational status**		
Not attended formal education	209	40.8
Grade 1–8	233	45.5
Grade 9 and above	70	13.7
**Estimated annual income** Median annual income = 8,000.00 EB (IQR = 5,000.00)		
<10,000	396	77.3
10,000–20,000	69	13.5
>20,000	47	9.2

EB = Ethiopian birr; IQR = Interquartile Range

### Experience of households in CBHI scheme

This study included households who had at least one family member that had fallen sick and visited health facilities. Accordingly, the majority (77.5%) of the respondents visited only health centers. A small proportion of participants (7.6%) visited only hospitals.

Over 80% of households had visited healthcare institutions more than twice after enrolment. The median length of enrollment was 23 months (IQR = 12 months).

Household members enrolled in CBHI scheme are required to visit public health facilities within the district or the nearest public hospitals with an agreement with the CBHI scheme. About 95.9% of household heads reported that they were happy with the permitted healthcare institutions. During their last visits to health care institutions, 442 (86.3%) of household heads reported that they received the correct prescribed drugs and 440 (85.9%) reported that they received the requested laboratory services. The majority of participants (86.3%) mentioned that they had participated in CBHI related meetings (Table 2).

**Table 2:** Experience of households in CBHI scheme in a piloted CBHI scheme in Sheko district, Southwest Ethiopia, 2018 (n = 512).

Variables	Frequency	Percent
**Length of enrollment** Median = 23.00 Months(IQR = 12)		
<12 months	158	30.9
≥12 months	354	69.1
**Health institution/facility visited**		
Only hospital	76	14.8
Both hospital and health center	39	7.6
Only health center	397	77.5
**Frequency of health facility visiting**		
Once	87	17.0
1–5 times	274	53.5
>5 times	151	29.5
**Happy with the permitted health institutions**		
Yes	491	95.9
No	21	4.1
**Participated in CBHI related meeting**		
Yes	442	86.3
No	70	13.7
**Got prescribed drugs**		
Yes	442	86.3
No	70	13.7
**Received required laboratory**		
Yes	440	85.9
No	72	14.1

### Household’s knowledge of CBHI benefit packages

Seven items were used to measure household’s knowledge of the CBHI benefit package. Households were labeled as having adequate knowledge if answered more than four of the CBHI benefit package. Otherwise, households were labeled as not having adequate knowledge of the CBHI benefit package. Accordingly, only 234 (45.7%) of the households had adequate knowledge of the CBHI benefit package (Table 3).

**Table 3:** Household’s knowledge of CBHI benefit packages in a piloted CBHI scheme in Sheko district, Southwest Ethiopia, 2018 (n = 512).

Variables	Frequency	Percent
**CBHI is good way of helping clients to health expenditure**		
Yes	488	95.3
No	24	4.7
**CBHI covers only care from public health institutions**		
Yes	463	90.4
No	49	9.6
**CBHI covers only care with in the country**		
Yes	497	97.1
No	15	2.9
**CBHI doesn’t cover transportation fee**		
Yes	92	18.0
No	420	82.0
**CBHI covers outpatient care**		
Yes	126	24.6
No	386	75.4
**CBHI covers inpatient care**		
Yes	380	74.2
No	132	25.8
**CBHI doesn’t cover medical care for cosmetic values**		
Yes	57	11.1
No	455	88.9
**Answered more than four of the CBHI benefit package**		
Yes	234	45.7
No	278	54.3

### Health service provision related characteristics of participants enrolled to CBHI scheme

Participants were asked four questions related to different aspects of health service provision. The majority of (87.9%) the participants mentioned that they agreed with laboratory services received and about 80.1% of them agreed that they got immediate care when visiting health facilities. However, nearly a quarter of (23.4%) the participants believed that they didn’t get respect from the health care providers (Table 4).

**Table 4:** Health service provision related characteristics of participants enrolled to CBHI in piloted Sheko district, Southwest Ethiopia, 2018 (n = 512).

Variables	Frequency	Percent
**Agreed with the laboratory services received**		
Agree	450	87.9
Disagree	62	12.1
**Got immediate care when visiting health facility**		
Agree	410	80.1
Disagree	102	19.9
**Got respect from health care providers**		
Agree	392	76.6
Disagree	120	23.4
**Healthcare service providers were friendly**		
Agree	396	77.3
Disagree	116	22.7

### Level of satisfaction with the CBHI scheme

Household satisfaction with CBHI scheme was rated using 9 items each having a five point Likert scale from strongly disagree to strongly agree. The internal consistency of the 9 items using cronbach’s alpha was 0.802. The points obtained from the 9 items by each respondent were computed to get the total score of each respondent. A respondent had a minimum of 18 and a maximum of 45 points on the CBHI scheme satisfaction score. Then, households were categorized as not satisfied (if they score below the median) or satisfied (if they score ≥ to the median satisfaction score).

The median score for household’s satisfaction on the CBHI scheme was 41.00. Based on our operational definition, about 280(54.7%) respondents scored ≥ to the median satisfaction score and the remaining 232(45.3%) scored below the median satisfaction score.

### Bivariate and multivariable analysis

Without controlling confounders, some of the socio-demographic and CBHI experience related variables were significantly associated with household’s satisfaction to CBHI scheme. At the bivariate level of analysis, age, educational status, length of enrolment, type and frequency of health facility visit were significantly associated with household’s satisfaction to CBHI scheme (p<0.05). Participants knowledge of CBHI benefit packages were significantly associated with household’s satisfaction to CBHI scheme (p<0.05). Furthermore, participants who agreed with the laboratory services received were more satisfied with CBHI scheme than who disagreed (p<0.2).

In the multivariable analysis, households who enrolled ≥12 months were 1.53 more satisfied to CBHI than households who enrolled <12 months (95% CI = (1.01–2.32). The likelihood of households satisfaction to CBHI was nearly twice among households who visited only hospitals compared to who visited only health centers (AOR = 1.93, 95% CI = (1.09–3.39). Participants who had adequate knowledge of CBHI benefit packages were 2.27 times more likely satisfied than who didn’t have adequate knowledge (95% CI = (1.55–3.38). Moreover, households satisfaction to CBHI scheme was 2.07 higher among households who agreed with the laboratory services received compared to who didn’t agree (95%CI = (1.15–3.73) (Table 5).

**Table 5:** Multivariable logistic regression analysis results on factors associated with overall household’s satisfaction in Sheko district, Southwest Ethiopia, 2018.

Variables	Satisfied	Not satisfied	COR with 95% CI	AOR with 95%CI
**Household head’s Age**				
<30 years	87(66.9%)	43(33.1%)	1.26(0.63–2.51)	1.19(0.56–2.53)
30–39 years	118(51.3%)	112(48.7%)	0.65(0.34–1.24)	0.63(0.32–1.25)
40–49 years	46(43.8%)	59(56.2%)	0.48(0.24–0.98)	0.49(0.23–1.03)
≥50 years	29(61.7%)	18(38.3%)	R	R
**Household head’s educational status**				
No formal education	96(45.9%)	113(54.1%)	0.44(0.25–0.78)	0.65(0.35–1.19)
Grade 1–8	138(59.2%)	95(40.8%)	0.76(0.43–1.32)	0.97(0.53–1.77)
Grade 9 and above	46(65.7%)	24(34.3%)	R	R
**Length of enrolment(in months)**				
<12	76(48.1%)	82(51.9%)	R	R
≥12	204(57.6%)	150(42.4%)	1.47(1.01–2.14)	1.53(1.01–2.32)*
**Health institution/facility visited**				
Only hospital	52(68.4%)	24(31.6%)	1.89(1.12–3.19)	1.93(1.09–3.39)*
Hospital and health center	16(41.0%)	23(59.0%)	0.61(0.31–1.18)	0.72(0.35–1.46)
Only health center	212(53.4%)	185(46.6%)	R	R
**Frequency of health facility visiting**				
Once	57(65.5%)	30(34.5%)	1.73(1.01–2.99)	1.38(0.76–2.53)
1–5 times	144(52.6%)	130(47.4%)	1.01(0.68–1.50)	0.95(0.60–1.48)
>5 times	79(52.3%)	72(47.7%)	R	R
**Agreed with the laboratory services received**				
Agree	251(55.8%)	199(44.2%)	1.43(0.84–2.44)	2.07(1.15–3.73)*
Disagree	29(46.8%)	33(53.2%)	R	R
**Adequate knowledge of CBHI benefit packages**				
Yes	152(65.0%)	82(35.0%)	2.17(1.52–3.11)	2.27(1.55–3.38)*
No	128(46.0%)	150(54.0%)	R	R

R reference category

* Significant at p value <0.05

## Discussion

CBHI may help to achieve the World Health Assembly call on all countries to move towards universal health coverage, especially in LICs with significant inequalities in health service delivery[19, 21]. Some LICs have started to implement the CBHI program. However, the quality of CBHI is poorly documented. Here, we assessed the perceived quality of CBHI scheme using client satisfaction in one of the piloted districts in rural Southwest Ethiopia.

We found a moderate level of household’s satisfaction to CBHI scheme. This finding is comparable with a study finding in Nigeria (42.1%) [17]. However, our finding is lower than the level of satisfaction reported in an earlier study in Ethiopia [18]. This divergence from earlier finding may be due to the difference in the definition of satisfaction. In the former study, the satisfaction score was calculated based on the percentage of maximum scale. Calculating satisfaction based on the percentage of maximum scale might overestimate the proportion of households satisfied to CBHI in the prior study. Alternatively, the high level of CBHI satisfaction in the former study may be due to improvements in the quality of health services. For instance, 98.2% of participants in the former study mentioned that the quality of health services was improved after the introduction of the CBHI scheme in the district[18].

Many health insurance satisfaction studies have tried to relate member’s satisfaction with socio-demographic variables. In this regard, several studies showed that enrollee’s satisfaction to the health insurance is significantly associated with socio-demographic variables such as marital status [16, 17], age [16, 18], gender [16], family size [18], and education status [16]. However, these studies didn’t control potential confounders such as enrollee’s knowledge of health insurance benefit packages [16, 18] and the quality of health care [16, 17]. In this study, after controlling potential confounders, socio-demographic and economic variables were not significantly associated with household’s satisfaction to CBHI scheme. A similar finding was reported in India, where the socio-demographic and economic variables such as age, gender, literacy level and economic status were not significantly associated with enrollee’s satisfaction to the health insurance scheme[14].

However, modifiable factors such as knowledge of the CBHI benefit packages, type of health facility visit, length of enrolment and variables related to the quality of services like laboratory services were significantly associated with household’s satisfaction to the CBHI scheme in this study area. We observed a moderate association between household’s satisfaction to CBHI and the type of health facility visit. Households who visited only hospitals were more satisfied compared to households who visited only health centers. This is in line with a study finding in Nigeria [17]. CBHI scheme enrollees may expect a high level of care from hospitals than health centers and their expectation might be met. Enrollees satisfaction and the decision to join in the health insurance is also depends on whether CBHI meets consumer’s expectations and needs [19]. In the Ethiopian context, hospitals are more equipped with doctors and facilities compared with health centers. When health insurance enrollees visit hospitals, they may more likely perceive that the available services and providers are appropriate for them. For example in India, Devadasan N and colleagues found that availability of doctors and medicines were the main reasons for client’s satisfaction to health insurance[14].

One of the health service provision related variables significantly associated with satisfaction was laboratory services. Participants who agreed with the laboratory services received were more satisfied compared to who disagreed. This finding is in line with a former study report in Ethiopia [18]. A significant association between the laboratory services received and CBHI satisfaction may suggest that the quality of health services also determine household’s satisfaction to CBHI scheme in this study area. However, as a limitation of this study (chicken egg dilemma), it is unknown whether the laboratory services received leads to satisfaction or their involvement in CBHI scheme itself increase expectation in laboratory services.

In this study, we found that CBHI enrollees with longer length of enrollment were more satisfied than enrollees with shorter length of enrollment. This finding is in line with a study finding in Nigeria [17]. Higher level of satisfaction among enrollees with longer length of enrollment may be explained in two ways:i) the quality of health care might be improved over time. As the quality care improves, enrollee’s satisfaction to CBHI scheme also increases. ii) CBHI enrollees may have lowered their expectations regarding the quality of care they received over time. When client’s expectation is minimal, they will be easily satisfied with the available health services. Further research is needed to ascertain which of the above justifications more likely explain the high level of satisfaction among CBHI enrollees with longer length of enrollment.

More importantly, household’s knowledge of the CBHI benefit packages was significantly associated with CBHI satisfaction. Poor knowledge of the health insurance package has affected the utilization of health insurance in LICs[19, 21]. CBHI enrollees may have a basic understanding of the role of CBHI but details about the benefit packages are not widely known[22]. Studies in sub-Saharan Africa showed that enrollee’s knowledge of the health insurance benefit package is poor and often associated with dropout [23, 24] and poor compliance[22]. Health insurance enrollee’s satisfaction gets better only if they understand how the health insurance scheme works and know what is being offered by the scheme [17]. Thus, effective behavioral education and communication need to be implemented to ensure that the health insurance benefit packages are understood by all enrollees.

### Limitation of the study

In this study, we have only explored enrollee satisfaction from the households (demand side). Providers such as CBHI cadres and health professional’s view were not explored.

## Conclusion

Household’s satisfaction to CBHI in the piloted Sheko district was moderate. Adequate knowledge of CBHI benefit packages, type of health facility visit, laboratory service provision, and length of enrollment were independent determinants of satisfaction. To further enhance enrollee’s satisfaction to CBHI scheme, efforts should be given to improving their knowledge of CBHI benefit packages through education and information campaigns. Furthermore, due consideration should also be given to improving the quality of health services.

## Supporting information

S1 File Survey questions in the English and original language. (DOCX)

## Abbreviations

AOR: Adjusted odd ratio
CBHI: Community-Based Health Insurance
COR: Crude odd ratio
IOR: Interquartile Range
LICs: Low-Income Countries
OOP: Out-of–Pocket
PPS: Probability Proportional to Sample size
SHI: Social Health Insurance
SPSS: Statistical Package for Social Science
SRS: Simple random Sampling
UHC: Universal Health Coverage
