# Supplementary material for: Household satisfaction with community-based health insurance scheme and associated factors in piloted Sheko district; Southwest Ethiopia
Source: PLoS One. 2019 May 13;14(5):e0216411. doi: 10.1371/journal.pone.0216411 (PMC6513074; doi:10.1371/journal.pone.0216411)
Supplement: S1 File — (DOCX) [file pone.0216411.s001.docx]

**Survey questions in the English and original language**

1. **English language**

| Number | Questions | Options | Code | Skip |
| --- | --- | --- | --- | --- |
| **Section1: Socio- demographic characteristics** | | | | |
| 1 | What is your age? | _______years (current age in years) |  |  |
| 2 | What is your sex? | 1. Male 2. Female | 1  2 |  |
| 3 | What is your current marital status? | 1. Single 2. Married with one spouse 3. Separated/divorced 4. Married with more than one 5. Widowed | 1  2  3  4  5 |  |
| 4 | How many household members are there in the household? | ________­­­­­­________numbers |  |  |
| 5 | What is your current religion? | 1. Orthodox 2. Protestant 3. Catholic 4. Muslim 5. Others(specify)_____________ | 1  2  3  4  5 |  |
| 6 | What is your current educational status? | 1. Not able to read and write 2. Able to read and write 3. Grade 1–8 4. Grade 9 and above | 1  2  3  4 |  |
| 7 | How much is your estimated annual income? | ____________________Birr |  |  |
| **Section 2: Experience with CBHI** | | | | |
| 8 | Who decided to enroll as a member of the CBHI scheme? | 1. Myself 2. Health care professionals 3. Kebele administrators 4. Health extension workers 5. Others(specify)_____________ | 1  2  3  4  5 |  |
| 9 | Length of enrolment | ________________ months |  |  |
| 10 | Which health institution did you visit? | 1. Health center 2. Hospital 3. Other(specify)_______________ | 1  2  3 |  |
| 11 | How many times did you visit? | 1. Only once 2. Two times 3. Three times 4. Four times 5. Five times 6. More than five times | 1  2  3  4  5  6 |  |
| 12 | According to the CBHI regulation, CBHI users are required to visit public health centers within the district/zone. Are you happy with the permitted health institutions? | 1. Yes 2. No | 1  2 |  |
| 13 | During the recent visit to the health care institutions, did the sick family members receive drugs? | 1. Yes 2. No | 1  2 |  |
| 14 | During the recent visit to the health care institutions, do you think that the sick family members received the correct prescribed drug? | 1. Yes 2. No | 1  2 |  |
| 15 | During the recent visit to the health care institutions, did the sick family members receive laboratory services? | 1. Yes 2. No | ^1^  ^2^ |  |
| 16 | During the recent visit to the health care institutions, do you think that the sick family members received the required laboratory services? | 1. Yes 2. No | 1  2 |  |
| 17 | Have you ever participated CBHI related meeting? | 1. Yes 2. No | 1  2 |  |
| **Section 3: Knowledge on CBHI package** | | | | |
| 18 | CBHI is good way of helping clients to relive health expenditure | 1. Yes 2. No | 1  2 |  |
| 19 | CBHI covers only care from public health institutions | 1. Yes 2. No | 1  2 |  |
| 20 | CBHI covers only care with in the country | 1. Yes 2. No | 1  2 |  |
| 21 | CBHI doesn’t cover transportation fee | 1. Yes 2. No | 1  2 |  |
| 22 | CBHI covers inpatient care | 1. Yes 2. No | 1  2 |  |
| 23 | CBHI covers outpatient care | 1. Yes 2. No | 1  2 |  |
| 24 | CBHI will no cover medical care for cosmetic values | 1. Yes 2. No | 1  2 |  |

**Section 4: Health services provision related questions**

| No | Questions | Options | Code | Skip |
| --- | --- | --- | --- | --- |
|  |  |  |  |  |
| 25 | Household members were satisfied with laboratory services | 1. Agree 2. Disagree | 1  2 |  |
| 26 | Household members got immediate care when visiting health facility | 1. Agree 2. Disagree | 1  2 |  |
| 27 | Household members got respect from health care providers | 1. Agree 2. Disagree | 1  2 |  |
| 28 | Health care service providers were friendly | 1. Agree 2. Disagree | 1  2 |  |

**Section 5: Questions related to satisfaction with CBHI**

| No | Questions | Strongly agree |  | Skip |
| --- | --- | --- | --- | --- |
| 29 | Household members are satisfied with the opening hours of the CBHI | 1. Strongly disagree 2. Disagree 3. Neutral 4. Agree 5. Strongly agree | 1  2  3  4  5 |  |
| 30 | Household members are satisfied with the collection process of insurance cards | 1. Strongly disagree 2. Disagree 3. Neutral 4. Agree 5. Strongly agree | 1  2  3  4  5 |  |
| 31 | Household members are satisfied with the time to make use of the CBHI program after payment of registration fee | 1. Strongly disagree 2. Disagree 3. Neutral 4. Agree 5. Strongly agree | 1  2  3  4  5 |  |
| 32 | Household members are satisfied with the schedule for paying of premium | 1. Strongly disagree 2. Disagree 3. Neutral 4. Agree 5. Strongly agree | 1  2  3  4  5 |  |
| 33 | Local CBHI management is trust worthy | 1. Strongly disagree 2. Disagree 3. Neutral 4. Agree 5. Strongly agree | 1  2  3  4  5 |  |
| 34 | Household members are satisfied with the information provided about CBHI | 1. Strongly disagree 2. Disagree 3. Neutral 4. Agree 5. Strongly agree | 1  2  3  4  5 |  |
| 35 | Household members satisfied with CBHI packages | 1. Strongly disagree 2. Disagree 3. Neutral 4. Agree 5. Strongly agree | 1  2  3  4  5 |  |
| 36 | Household members stay enrolled in the CBHI scheme | 1. Strongly disagree 2. Disagree 3. Neutral 4. Agree 5. Strongly agree | 1  2  3  4  5 |  |
| 37 | CBHI need to be scale up to other districts | 1. Strongly disagree 2. Disagree 3. Neutral 4. Agree 5. Strongly agree | 1  2  3  4  5 |  |

**Thank you!**

**2. Amharic language**

**ቀበሌ:**_________________

**መንደር:**_________________

**የቤት ቁጥር (ኮድ)**_________

| ተ.ቁ | ጥያቄዎች | መልሶች | ኮድ | ዝለል |
| --- | --- | --- | --- | --- |
| **ክፍል 1: አጠቃላይ መረጃ** | | | | |
| 1 | የአባወራ/ የእማወራ እድሜ | ---------------ዓመት |  |  |
| 2 | የአባወራ/የእማወራ ፆታ | 1. ወንድ  2. ሴተ | 1  2 |  |
| 3 | የአሁን ወቅት የጋብቻ ሁኔታ | 1-ያላገባ  2-ያገባ(አንድ ብቻ)  3-ያገባ(ከአንድ በላይ)  4-የፈታ (የፈታች)  5-ቧሏ የሞተባት/ሚስቱ የሞተችበት | 1  2  3  4  5 |  |
| 4 | የቤተሰብ ብዛት | -----------በቁጥር |  |  |
| 5 | የአባወራው/ የእማወራው  ሀይማኖት | 1-ኦርቶዶክስ  2-ፕርቶቴስታንት  3-ካቶሊክ  4-ሙስሊም  5-ሌላ(ይገለጽ)------------ | 1  2  3  4  5 |  |
| 6 | የአባወራው/ የእወራው  የትምርት ደረጃ | 1-ማንበብ ና መጻፍ የማይችል  2-ማንበብ ና መጻፍ የሚችል  3-ከ 1-8 የተማረ  4-ከ 9-12 የተማረ  5-ከ 12 በላይ | 1  2  3  4  5 |  |
| 7 | አመታዊ ገቢ | --------------ብር |  |  |
| **ክፍል 2: የማህበረሰብ አቀፍ የጤና መድን በተመለከተ** | | | | |
| 8 | የማህበረሰብ አቀፍ የጤና መድን እንዲመዘገቡ ያረገዎት ማነው | 1.እኔ እራሴ  2.የጤና ባለሙያ  3.የቀበሌ አስተዳደር  4. የጤና ኤክስቴንሽን ባለሙያ  5.ሌላ(ይገለጽ) ----------- | 1  2  3  4  5 |  |
| 9 | ከተመዘገቡ/አባል ከሆኑ ምን ያክል ግዜ ሆኖታል | ------------ወር |  |  |
| 10 | የትኛው የጤና ተቋም ነው የሄዱት | 1.ጤና ጣቢያ  2.ሆስፒታል  3. ሌላ(ይገለጽ)----------- | 1  2  3 |  |
| 11 | ለምን ያክል ጊዜ ነው ወደ ጤና ተቋም የሄዱት | 1. አንድ ጊዜ ብቻ 2. ሁለት ጊዜ 3. ሶስት ጊዜ 4. አራት ጊዜ 5. አምስት ጊዜ 6. ከአምስት ጊዜ በላይ | 1  2  3  4  5  6 |  |
| 12 | በማህበረሰብ አቀፍ የጤና መድን ህግ መሰረት በአከባቢዎ ባሉት የመንግስት ጤና ጣቢያዎች መጀመሪያ እንዲጎበኙ እና እንዲታከሙ ይጠበቅበዎታል፡፡ በዚህ ደስተኛ ነዎት | 1-አዎ  2-አይደለሁም | 1  2 |  |
| 13 | በቅርብ ጊዜ ከቤተሰብ የታመመ ሰዉ ወደ ጤና ተቋም በሄዱበት ወቅት መድሀኒት ተሰጥቶቹሃል | 1 አወ  2 አልተሰጠም | 1  2 |  |
| 14 | በቅርብ ጊዜ ከቤተሰብ የታመመ ሰዉ ወደ ጤና ተቋም በሄዱበት ወቅት ትክክለኛ እና የታዘዘለዎተን መድሀኒት አገኝተዋል ብለው ያስባሉ | 1.አዎ  2.አላስብም | 1  2 |  |
| 15 | በቅርብ ጊዜ ከቤተሰብ የታመመ ሰዉ ወደ ጤና ተቋም በሄዱበት ወቅት የላብራቶሪ ምርምራ ተደርጎላቸዋል | 1 አወ  2 አልተሰጠም | 1  2 |  |
| 16 | በቅርብ ጊዜ ከቤተሰብ የታመመ ሰዉ ወደ ጤና ተቋም በሄዱበት ወቅት የሚፈልጉትን የላብራቶሪ ምርምራ አግኝተዋል ብለው ያስባሉ | 1.አዎ  2. አላስብም | 1  2 |  |
| 17 | ከማህበረሰብ አቀፍ የጤና መድን ጋር የሚገናኝ ስብሰባ ተሰብስበው ያውቃሉ | 1. አዎ 2. የለም | 1  2 |  |
| **ክፍል 3: የማህበረሰብ አቀፍ የጤና መድን እዉቀትን በተመለከተ** | | | | |
| 18 | የማህበረሰብ አቀፍ የጤና መድን የጤና ወጭን የምንቀንስበት ጥሩ መንገድ ነው | 1-አወ  2-አይደለም | 1  2 |  |
| 19 | የማህበረሰብ አቀፍ የጤና መድን አገልግሎት የሚያካትታቸው የመንግስት ተቋማትን አገልግሎት ብቻ ነው | 1-አወ  2-አይደለም | 1  2 |  |
| 20 | የማህበረሰብ አቀፍ የጤና መድን አገልግሎት የሚያካትታቸው የሀገር ውስጥ ህክምብን ብቻ ነዉ | 1-አወ  2-አይደለም | 1  2 |  |
| 21 | የማህበረሰብ አቀፍ የጤና መድን የትራንስፖርት (የመጓጓዣ) ወጭን አይሸፍንም | 1-አይሸፍንም  2-ይሸፍናል | 1  2 |  |
| 22 | የማህበረሰብ አቀፍ የጤና መድን ተኝተዉ ለሚታከሙ ወጭን ይሸፍናል | 1-ይሸፍናል  2-አይሸፍንም | 1  2 |  |
| 23 | የማህበረሰብ አቀፍ የጤና መድን የተመላላሽ ታካሚዎችን ወጭ ይሸፍናል | 1-ይሸፍናል  2-አይሸፍንም | 1  2 |  |
| 24 | የማህበረሰብ አቀፍ የጤና መድን የውበት ህክምና ወጭን አይሸፍንም | 1-አይሸፍንም  2-ይሸፍናል | 1  2 |  |

**ክፍል 4: ከጤና አገልገሎት ጋርየተገናኙ ጥያቄዎች**

| 25 | እኔ/ቤተሰቤ ጤና ተቋም ሂደን ባገኘነዉ የላብራቶሪ አገልግሎት እረክቻለሁ | 1. እስማማለሁ 2. አልስማማም | 1  2 |  |
| --- | --- | --- | --- | --- |
| 26 | እኔ/ቤተሰቤ ጤና ተቋም በሄድኩበት/በሄድንበት ጊዜ በአፋጣኝ አገልግሎት አግኝቻለሁ | 1. እስማማለሁ 2. አልስማማም | 1  2 |  |
| 27 | እኔ/ቤተሰቤ ጤና ተቋም በሄድንበት ጊዜ የጤና ባለሙያዎች ክብር አድርገውልን ነበር(በክብር አስተናግደውናል) | 1. እስማማለሁ 2. አልስማማም | 1  2 |  |
| 28 | የጤና አገልግሎት የሚሰጡ የጤና ባለሙያዎች እንደወንድምና እንደ እህት ያቀርቡን ነበር /ያነጋግሩን ነበር | 1. እስማማለሁ 2. አልስማማም | 1  2 |  |
| **ክፍል 5: የማህበረሰብ አቀፍ የጤና መድን እርካታን በተመለከተ** | | | |  |
| 29 | የማህበረሰብ አቀፍ የጤና መድን አገልግሎት በሚከፈትበት/ በሚጀመርበት ሰአት ደስተኛ ነን | 1.በጣም እስማማለሁ  2.እስማማለሁ  3.ሀሳብ የለኝም  4.አልስማማም  5.በጣም አልስማማም | 1  2  3  4  5 |  |
| 30 | በማህበረሰብ አቀፍ የጤና መድን ካርድ አሰጣጥ ሂደት ላይ ደስተኛ ነን | 1.በጣም እስማማለሁ  2.እስማማለሁ  3.ሀሳብ የለኝም  4.አልስማማም  5.በጣም አልስማማም | 1  2  3  4  5 |  |
| 31 | የምዝገባ ክፍያ ከከፈልን በኃላ የማህበረሰብ አቀፍ የጤና መድን አገልግሎት ለመጠቀም በወሰደው/በፈጀዉ ጊዜ ደስተኛ ነን | 1.በጣም እስማማለሁ  2.እስማማለሁ  3.ሀሳብ የለኝም  4.አልስማማም  5.በጣም አልስማማም | 1  2  3  4  5 |  |
| 32 | በማህበረሰብ አቀፍ የጤና መድን ገንዘብ ክፍያ መርሀ ግብር ደስተኛ ነን | 1.በጣም እስማማለሁ  2.እስማማለሁ  3.ሀሳብ የለኝም  4.አልስማማም  5.በጣም አልስማማም | 1  2  3  4  5 |  |
| 33 | በአከባቢያችን ያለው የማህበረሰብ አቀፍ የጤና መድን አስተዳደር እምነት የሚጣልበት ነው | 1.በጣም እስማማለሁ  2.እስማማለሁ  3.ሀሳብ የለኝም  4.አልስማማም  5.በጣም አልስማማም | 1  2  3  4  5 |  |
| 34 | በማህበረሰብ አቀፍ የጤና መድን ዙሪያ በሚሰጠው መረጃ ተደስተናል | 1.በጣም እስማማለሁ  2.እስማማለሁ  3.ሀሳብ የለኝም  4.አልስማማም  5.በጣም አልስማማም | 1  2  3  4  5 |  |
| 35 | የማህበረሰብ አቀፍ የጤና መድን በያዛቸው ፖኬጆች (ጥቅል አገልግሎቶች) ተደስተናል | 1.በጣም እስማማለሁ  2.እስማማለሁ  3.ሀሳብ የለኝም  4.አልስማማም  5.በጣም አልስማማም | 1  2  3  4  5 |  |
| 36 | የማህበረሰብ አቀፍ የጤና መድን አባል ሆኖ መቆየት ያስደስተናል | 1.በጣም እስማማለሁ  2.እስማማለሁ  3.ሀሳብ የለኝም  4.አልስማማም  5.በጣም አልስማማም | 1  2  3  4  5 |  |
| 37 | የማህበረሰብ አቀፍ የጤና ኢንሹራንስ ወደ ሌላ ወረዳዎች መስፋፋት አለበት | 1.በጣም እስማማለሁ  2.እስማማለሁ  3.ሀሳብ የለኝም  4.አልስማማም  5.በጣም አልስማማም | 1  2  3  4  5 |  |

**አመሰግናለሁ!**
